# Treatment of renal fibrosis by rebalancing TGF-β/Smad signaling with the combination of asiatic acid and naringenin

**DOI:** 10.18632/oncotarget.6100

**Published:** 2015-10-12

**Authors:** Xiao-ming Meng, Yun Zhang, Xiao-Ru Huang, Gui-ling Ren, Jun Li, Hui Yao Lan

**Affiliations:** ^1^ Department of Medicine and Therapeutics, Li Ka Shing Institute of Health Sciences, Shenzhen Research Institute, The Chinese University of Hong Kong, Hong Kong SAR; ^2^ School of Pharmacy, Anhui Medical University, An Hui, China; ^3^ Department of Dermatology, Foshan Hospital of TCM, Foshan, China

**Keywords:** treatment for fibrosis, TGF-β/Smads, asiatic acid, naringenin, Pathology section

## Abstract

We recently showed that imbalance of TGF-β/Smad signaling with over-activation of Smad3 but lower levels of Smad7 is a central mechanism of tissue fibrosis. In the present study, we report here that inhibition of Smad3 with naringenin (NG) and upregulation of Smad7 with asiatic acid (AA) produced an additive effect on inhibition of renal fibrosis in a mouse model of obstructive nephropathy. We found that AA, a triterpene from Centella Asiatica, functioned as a Smad7 agonist and suppressed TGF-β/Smad3-mediated renal fibrosis by inducing Smad7. Whereas, NG, a flavonoid from grapefruits and citrus fruits, was a Smad3 inhibitor that inhibited renal fibrosis by blocking Smad3 phosphorylation and transcription. The combination of AA and NG produced an additive effect on inhibition of renal fibrosis by blocking Smad3 while upregulating Smad7. Thus, rebalancing the disorder of TGF-β/Smad signaling by treatment with AA and NG may represent as a novel and effective therapy for chronic kidney disease associated with fibrosis.

## INTRODUCTION

Renal fibrosis, characterized by the accumulation of myofibroblasts and extracellular matrix (ECM), is a major cause of the end-stage renal disease. However, treatment of renal fibrosis remains non-specific and ineffective clinically. Increasing evidence shows that TGF-β1 is a key mediator of renal fibrosis [1-5]. Many studies have attempted to develop anti-fibrosis therapy by inhibiting the upstream of TGF-β signaling, including antisense TGF-β oligodeoxynucleotides, neutralizing antibodies, and inhibitors to TGF-β receptor (TβR) kinases in a variety of kidney disease models [2, 6-8]. However, treatment of puromycin aminonucleoside nephropathy with a high dose of anti-TGF-β antibody produces no renoprotective effect [9], which is consistent with a recent finding that disruption of TGF-β type II receptor decreased renal fibrosis but enhanced renal inflammation [10]. Results from these studies suggest that inhibiting the upstream of TGF-β signaling may not be an optimal therapeutic approach due to the diverse roles of TGF-β1 in inflammation and fibrosis. Although blockade of the upstream TGF-β signaling inhibits fibrosis, it may also promote inflammation. Therefore, we hypothesized that treatment of renal fibrosis should target the downstream TGF-β/Smad signaling associated with fibrosis, rather than to block the general effect of TGF-β1.

Increasing evidence shows that TGF-β/Smad signaling is a central pathway leading to renal fibrogenesis [1]. It is now clear that TGF-β1 promotes fibrosis positively by activating its downstream molecule Smad3, not Smad2, but negatively by an inhibitory Smad7 [2, 11]. In the context of fibrosis, Smad3 is pathogenic, which is supported by the finding that mice lacking Smad3 are protected against tissue fibrosis in chronic kidney and cardiac diseases [12-15]. In contrast, Smad7 is protective during fibrosis as deletion of Smad7 promotes, but overexpression of Smad7 inhibits, tissue fibrosis [16-19]. Thus, the imbalance of TGF-β/Smad signaling may be a major cause of renal fibrosis and rebalancing this pathway by inactivating Smad3 while upregulating Smad7 may produce a better therapeutic effect on renal fibrosis.

Asiatic Acid (AA), a triterpenoid component extracted from Centella asiatica [20], has been shown to have a variety of pharmacological effects including anti-inflammation [21], anti-oxidation [22] and anti-fibrosis [23, 24]. We recently found that AA exerts its anti-fibrotic effects on liver fibrosis by inducing Smad7 [23]. Naringenin (NG) is a flavonoid from grapefruit and citrus fruits with anti-inflammatory properties in a number of disease conditions including atherosclerosis and diabetes [25-27]. NG is also found to inhibit TGF-β/Smad3 signaling and tissue fibrosis including epithelial-mesenchymal transition in a number of disease models [28, 29]. Based on these observations, we therefore hypothesized that the combination of AA and NG may produce a better therapeutic effect on renal fibrosis by more effectively correcting the imbalance of TGF-β/Smad signaling, namely upregulation of Smad7 while suppressing Smad3. The hypothesis was examined in the present study *in vitro* and in a well-characterized mouse model of unilateral ureteral obstruction (UUO).

## RESULTS

### Combination of AA and NG produces a better effect on restoring the balance of TGF-β/Smad signaling and inhibiting TGF-β1-induced fibrosis *in vitro*

**Figure 1:**
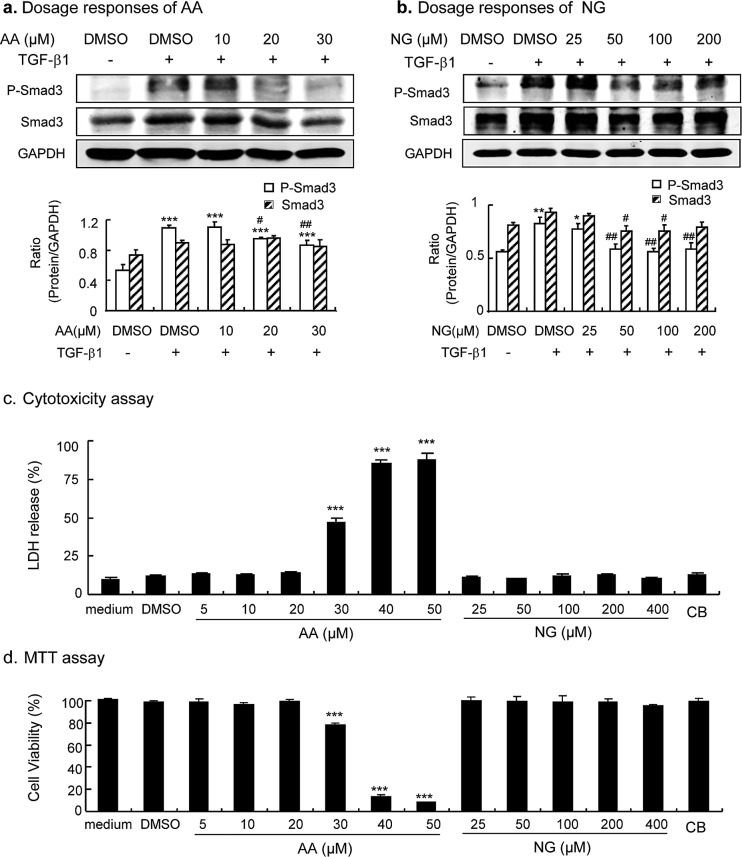
Dose-dependent effect of AA, NG, and their combination on inhibition of TGF-β/Smad3 signaling and cytotoxicity in cultured TECs **a.** Dose-dependent effect of AA on TGF-β1(2ng/ml)-induced Smad3 phosphorylation and protein expression. **b.** Dose-dependent effect of AA on TGF-β1(2ng/ml)-induced Smad3 phosphorylation and expression. **c.** Dose-dependent effect of AA, NG and their combination on cytotoxicity as determined by the LDH release assay. Data represent the mean ± SEM for at least 3 independent experiments. **d.** Dose-dependent effect of AA, NG and their combination on cytotoxicity as determined by the MTT assay. *p < 0.05, *p* < 0.01, ***p < 0.001 compared to the DMSO control. ^#^*p* < 0.05, ^##^*p* < 0.01 compared to DMSO + TGF-β1 treatment.

We first determined an effective and safe dosage of AA and NG *in vitro*, in which a better effect on restoring the balance of TGF-β/Smad signaling and inhibiting fibrosis without cytotoxicity was achieved. As shown in Figure 1A, phosphorylation of Smad3 was significantly induced in renal tubular epithelial cells (TECs) at 30 minutes after addition of TGF-β1 (2ng/ml), which was significantly inhibited by over-night pre-incubation with AA at dosages of 20 μM and 30μM (Figure 1a). Similarly, addition of NG was also able to block TGF-β1-induced activation of Smad3 in a dosage-dependent manner, with significant dosages over 50μM (Figure 1b). We then examined the cytotoxic response to AA or NG in cultured TECs. As shown in Figure 1c and 1d, lactate dehydrogenase (LDH) releasing assay and MTT assay detected that AA at concentrations over 30μM caused significant cytotoxicity and largely decreased cell viability of TECs. In contrast, the cytotoxic and anti-proliferation effects of NG were undetectable with the concentration range from 25μM to 400μM (Figure 1c and 1d). Taken together, AA at 20μM and NG at 50μM were selected as an effective and safe dosage for the *in vitro* study. Furthermore, the combination of AA (20μM) and NG (50μM) also produced no cytotoxicity to TECs (Figure 1c).

**Figure 2:**
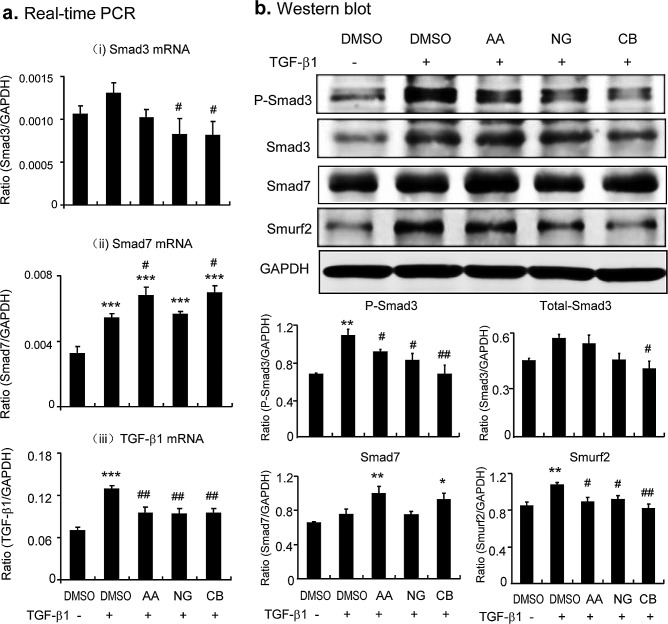
Combination of AA and NG produces an additive effect on inhibition of TGF-β/Smad signaling via differential mechanisms *in vitro* **a.** Effect of AA, NG, and their combination (CB) on TGF-β1(2ng/ml)-induced mRNA expression of Smad3, Smad7, and TGF-β1 by real-time PCR. **b.** Effect of AA, NG, and their combination (CB) on TGF-β1(2ng/ml)-induced protein levels of phospho-Smad3, Smad3, Smad7, and Smurf2 by Western blot analysis. Note that while AA inhibits phosphorylation of Smad3 by induction of Smad7, NG blocks Smad3 signaling by inhibiting Smad3 mRNA and protein expression. The combination of AA and NG additively inhibits TGF-β/Smad3 signaling. Data represent the mean ± SEM for at least 3 independent experiments. *p < 0.05, *p* < 0.01, ***p < 0.001 compared to the DMSO control. ^#^*p* < 0.05, ^##^*p* < 0.01 compared to DMSO + TGF-β1 treatment.

After determining an effective and safe dosage of AA, NG, and their combination use, the mechanisms of drugs actions were examined in TGF-β1-stimulated TECs. Real-time PCR and Western blot showed that addition of AA induced upregulation of Smad7 at both mRNA and protein levels, which was associated with inhibition of Smad3 phosphorylation but not expression (Figure 1a, Figure 2). These observations suggested that AA acts by inducing Smad7 to inhibit TGF-β1-induced Smad3 signaling. Similarly, addition of NG was capable of blocking TGF-β1-induced phosphorylation of Smad3 (Figs.1b and Figure 2b). Interestingly, NG also inhibited expression of Smad3 in both mRNA and protein levels (Figure 2); however, treatment with NG failed to induce Smad7 transcription (Figure 2a ii), revealing that NG inhibits TGF-β/Smad3 signaling by blocking Smad3 phosphorylation and transcription.

**Figure 3:**
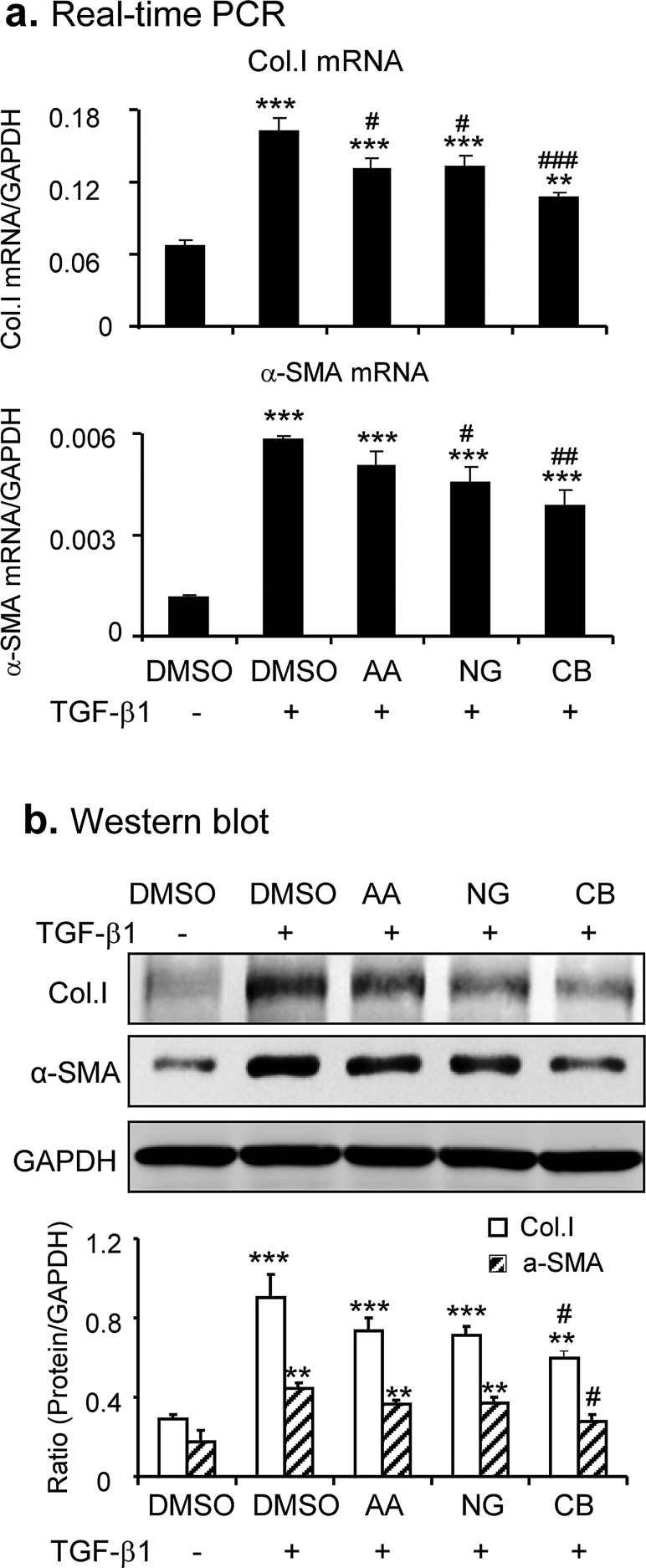
Combination of AA and NG produces a better protective effect on TGF-β1-induced fibrotic response *in vitro* **a.** Effect of AA, NG, or their combination on TGF-β1 (2ng/ml)-induced mRNA expression of collagen I and α-SMA detected by real-time PCR. **b.** Effect of AA, NG, or their combination on TGF-β1 (2ng/ml)-induced mRNA expression of collagen I and α-SMA detected by Western blot analysis. Data represent the mean ± SEM for at least 3 independent experiments. **p < 0.01, ***p < 0.001 compared to the DMSO control. ^#^*p* < 0.05, ^##^*p* < 0.01, ^###^*p* < 0.001 compared to DMSO + TGF-β1 treatment.

Because AA and NG acted by different mechanisms to inhibit TGF-β/Smad signaling, we tested if the combination of AA and NG produces a better inhibitory effect on TGF-β/Smad signaling and fibrosis. As shown in Figure 2, although the combination of AA (20μM) and NG (50μM) produced little additive effect on mRNA levels of Smad3 or Smad7 (Figure 2a i, ii), TGF-β1-induced phosphorylation of Smad3 and expression of Smurf2 were additively suppressed (Figure 2b). Real-time PCR and Western blot revealed that the combined treatment with AA and NG also produced an additive inhibitory effect on TGF-β1-induced collagen I and α-SMA expression in TGF-β1-stimulated TECs (Figure 3). However, the combination of AA and NG produced no additive effect on inhibition of TGF-β1 mRNA expression (Figure 2a iii).

### Combination treatment with AA and NG produces a better anti-fibrotic effect on UUO nephropathy *in vivo*

**Figure 4:**
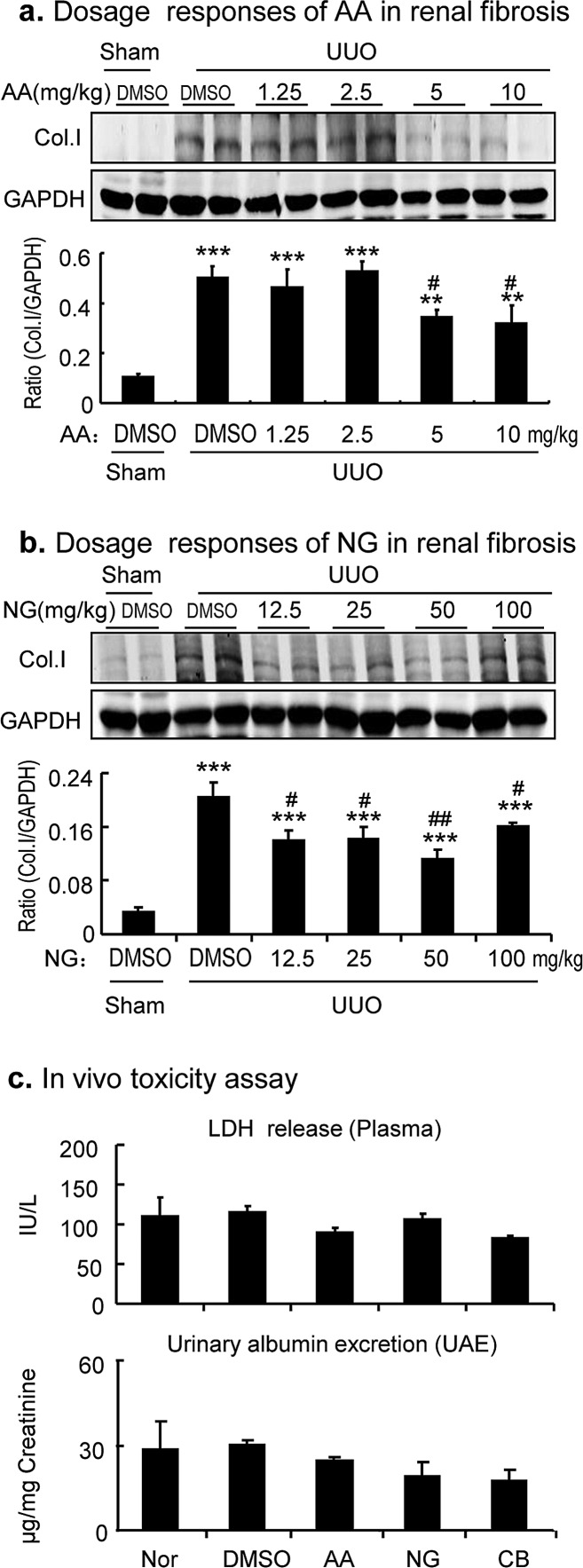
Dose-dependent effect of AA, NG, and their combination on inhibition of renal fibrosis in a mouse model of UUO **a.** Dose-dependent responses of AA in inhibition of collagen I expression by Western blot analysis. **b.** Dose-dependent responses of NG in inhibition of collagen I expression by Western blot analysis. **c.** LDH release assay for determination of the toxicity of AA (5mg/kg), NG (50mg/kg), and their combination in groups of 4 normal mice. Data represent the mean ± SEM for groups of 4 mice. **p < 0.01, ***p < 0.001 compared to sham-operated mice. ^#^*p* < 0.05, ^##^*p* < 0.01 compared to DMSO-treated UUO mice.

We next examined if the combination of AA and NG also produced a better inhibitory effect on renal fibrosis in a well-established mouse model of UUO. We first determined an optimal dosage of AA and NG and their combination for a better anti-fibrosis effect without toxicity in a mouse model of UUO. As shown in Figure 4, daily intraperitoneal injection (i.p.) of AA for 7 days was able to inhibit collagen I expression in the UUO kidney in a dose-dependent manner, with a better inhibitory effect at dosages of 5 and 10 mg/kg body weight (BW). In addition, i.p. injection of NG also dose-independently inhibited collagen I production in the UUO kidney, with an optimal dosage at 50 mg/kg BW. Thus, AA at 5mg/kg BW and NG at 50 mg/kg BW were selected as a single or combination treatment for renal fibrosis in a mouse model of UUO.

To determine the potential toxicity of selected dosages of AA, NG, and their combination *in vivo*, we treated groups of 4 normal mice with AA (5mg/kg BW), NG (50mg/kg BW), and their combination for 7 days and collected serum for LDH release assay and 24-hour urine samples for measuring urinary albumin excretion. As shown in Figure 4c, serum levels of LDH and urinary albumin concentrations were not altered after treatment with AA, NG, and their combination. In addition, no histological abnormalities were noticed based on H&E and PAS-staining (no shown).

**Figure 5:**
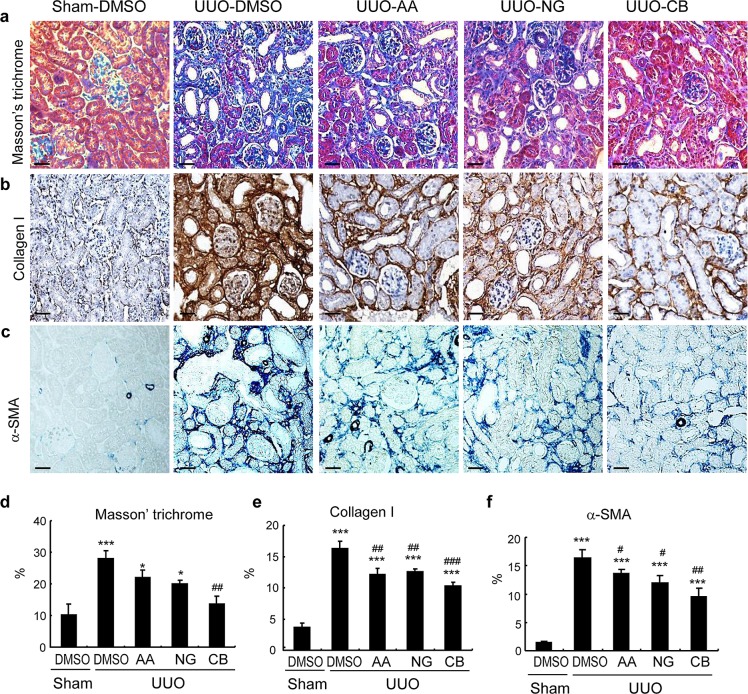
Histologic and immunohistochemical examination show that the combination treatment with AA and NG produces a better inhibitory effect on renal fibrosis in a mouse model of UUO **a.** Masson's trichrome staining. **b.** Immunohistochemical staining of collagen I. **c.** Immunohistochemical staining of α-SMA. **d.** Quantitative analysis of Masson trichrome staining. **e.** Quantitative analysis of collagen I immunostaining. **f.** Quantitative analysis of α-SMA Immunostaining. Data represent the mean ± SEM for groups of 6-8 mice. *p < 0.05, ***p < 0.001 compared to sham-operated mice. ^#^*p* < 0.05, ^##^*p* < 0.01 compared to DMSO-treated UUO mice. Scale bars, 50μM.

**Figure 6:**
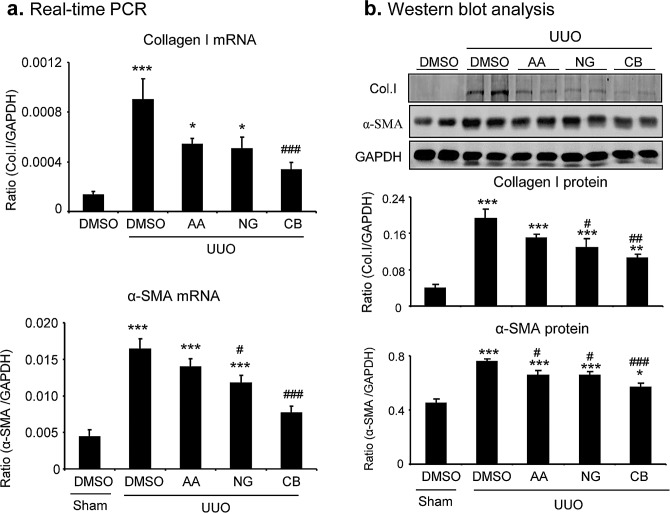
Real-time PCR and Western blot analysis detect that the combination treatment with AA and NG produces a better inhibitory effect on renal fibrosis in a mouse model of UUO **a.** Collagen I and α-SMA mRNA expression by real-time PCR. **b.** Collagen I and α-SMA protein expression by Western blotting. Results show that the combination of AA and NG treatment produces an additive effect on inhibition of collagen I and α-SMA expression. Data represent the mean SEM for groups of 6-8 mice. *p < 0.05, **p < 0.01, ***p < 0.001 compared to sham-operated mice. ^#^*p* < 0.05, ^##^*p* < 0.01, ^###^*p* < 0.001 compared to DMSO-treated UUO mice.

We then determined the therapeutic efficacy of AA, NG, and their combination in a mouse model of UUO as described above. Masson trichrome staining showed that single treatment with AA or NG significantly inhibited total collagen-like matrix deposition in the injured kidney, which was further improved by the combination treatment (Figure 5a and 5d). Immunohistochemistry also revealed that the combination treatment with AA and NG further decreased the accumulation of collagen I and α-SMA^+^ myofibroblasts within the UUO kidney when compared with the individual treatment alone (Figure 5b, 5c, 5e, 5f). This was further confirmed at the mRNA levels by real-time PCR and at the total kidney protein levels by western blot analysis (Figure 6).

### Mechanisms of AA, NG, and their combination treatment in renal fibrosis *in vivo*

**Figure 7:**
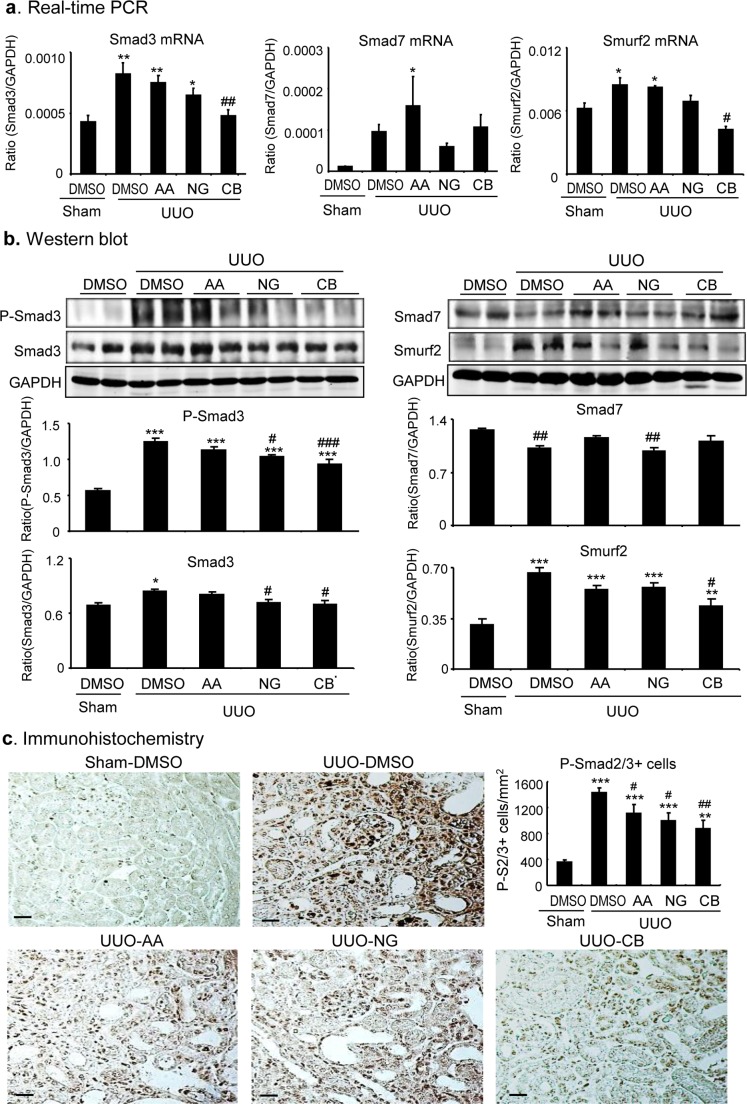
Combination treatment with AA and NG results in a further inhibition of TGF-β/Smad signaling in the UUO kidney **a.** Inhibitory effect of AA, NG, and their combination (CB) on Smad3, Smad7, and Smurf2 mRNA expression by real-time PCR. **b.** Western blot analysis shows the inhibitory effect of AA, NG, and their combination (CB) on phosphorylation levels of Smad3 and total protein levels of Smad3, Smad7, and Smurf2 in the UUO kidney. **c.** Immunohistochemistry shows the inhibitory effect of AA, NG, and their combination (CB) on phospho-Smad3 nuclear translocation in the UUO kidney. Note that while AA inhibits phosphorylation of Smad3 by induction of Smad7, NG blocks Smad3 signaling by inhibiting Smad3 mRNA and protein expression. The combination of AA and NG additively inhibits Smurf2 and TGF-/Smad3 signaling. Data represent the mean ± SEM for groups of 6-8mice. *p < 0.05, **p < 0.01, ***p < 0.001 compared to sham-operated mice. ^#^*p* < 0.05, ^##^*p* < 0.01 compared to DMSO-treated UUO mice. Scale bars, 50μM.

**Figure 8:**
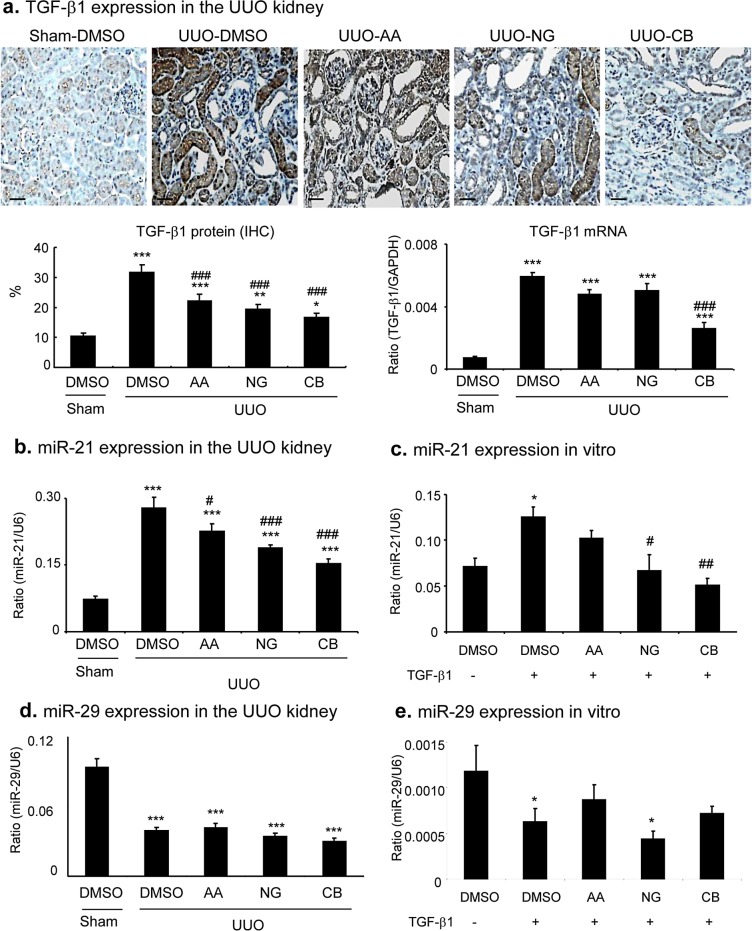
Combination treatment with AA and NG produces a further suppressive effect on renal TGF-β1 and miR-21 expression in the UUO kidney **a.** Immunohistochemistry and real-time PCR show the inhibitory effect of AA, NG, and their combination (CB) on TGF-β1 expression in the UUO kidney. **b.**, **c.** Real-time PCR detects that the combination treatment with AA and NG results in a further suppression of miR-21 expression in the UUO kidney and in cultured TECs. **d.**, **e.** Real-time PCR detects miR-29b expression in the UUO kidney and in cultured TECs. Data represent the mean ± SEM for groups of 6-8 mice. *p < 0.05, **p < 0.01, ***p < 0.001 compared to sham-operated mice. ^#^*p* < 0.05, ^##^*p* < 0.01 compared to DMSO-treated UUO mice. Scale bars, 50μM.

We next examined the mechanisms of AA, NG, and their combination in anti-renal fibrosis. As shown in Figures 7 and 8, treatment with AA or NG alone partially inhibited upregulation of TGF-β1 and phosphorylation of Smad3 and phospho-Smad3 nuclear translocation, which was further suppressed by the combination of AA and NG. Consistent with the findings *in vitro*, the inhibitory effect of AA on Smad3 signaling in the UUO kidney was correlated with a significant upregulation of renal Smad7 at both mRNA and protein levels without altering Smad3 mRNA and protein expression (Figure 7). In contrast, blockade of Smad3 signaling with NG was associated with downregulation of Smad3 at both mRNA ad protein levels without altering Smad7 mRNA and protein levels (Figure 7). The combination treatment with AA and NG produced a better inhibitory effect on TGF-β/Smad signaling by further suppressing Smad3 signaling while inducing Smad7 mRNA expression as well as preventing Smad7 protein from degradation by decreasing Smurf2 (Figure 7a, 7b).

Consistent with our previous finding that there was a markedly upregulation of miR-21 but downregulation of miR-29b in the UUO kidney [38, 39]. Interestingly, treatment with either AA or NG significantly reduced miR-21 expression, which was further suppressed when animals were treated with the combination of AA and NG (Figure 8b). A similar result was also found in the cultured TECs in which the combination treatment with AA and NG resulted in further inhibition of miR-21 expression (Figure 8c). However, treatment with AA, NG or the combination did not alter the expression of miR-29b in the UUO kidney and *in vitro* (Figure 8d).

## DISCUSSION

Increasing evidence shows that TGF-β1/Smad signaling is a central pathway leading to tissue fibrosis [1, 2]. It is now clear that Smad3 is a key fibrogenic mediator and is highly activated during renal fibrosis. In contrast, Smad7, an inhibitor of TGF-β/Smad3 signaling, is lost in the scarring kidney [2]. In the present study, we identified that AA, a purified compound from Centella asiatica [20], is a Smad7 agonist, whereas NG, a flavonoid from grapefruit and citrus fruits [28], functioned as a Smad3 inhibitor. The combination of these two purified Traditional Chinese Medicine compounds significantly enhanced the inhibitory effect on TGF-β/Smad signaling and renal fibrosis *in vitro* and *in vivo*. Outcomes from this study suggested that restoring the balance of downstream TGF-β/Smad signaling with AA (a Smad7 agonist) and NG (a Smad3 inhibitor) may represent a new therapeutic approach for chronic kidney disease associated with progressive renal fibrosis.

The identification of NG as a Smad3 inhibitor and AA as a Smad7 agonist was an important finding in the present study. Consistent with the consensus that Smad3 stimulates tissue fibrosis in a number of chronic kidney diseases [12, 13], inhibition of Smad3 transcription and phosphorylation was a mechanism through which NG inhibits fibrosis *in vitro* and *in vivo*. This finding was consistent with other studies in which inhibition of Smad3 with SIS3 (a Smad3 inhibitor to block Smad3 DNA binding and phosphorylation) and GQ5 (a small compound isolated from the dried resin of Toxicodendron vernicifluum that blocks the binding of Smad3 to TGF-β type I receptor) attenuates renal fibrosis [30, 31]. Interestingly, addition of NG blocks Smad3 mRNA and protein expression without altering levels of Smad7 expression, indicating a specific effect of NG on inhibiting Smad3 without influencing Smad7. In the present study, we also found that addition of AA was capable of blocking Smad3 phosphorylation and renal fibrosis by upregulating Smad7 without altering expression of Smad3, identifying AA as a Smad7 agonist to inhibit Smad3 signaling by inducing Smad7 transcription. This finding was consistent with our previous study that treatment with AA inhibits liver fibrosis by inducing Smad7 [23]. It is well accepted that Smad7 is an inhibitor of Smad3 signaling and protects against tissue fibrosis in a number of diseases [17, 32-35]. However, Smad7 is lost in the fibrosing tissue, which is induced by Smurf2, an E3 ligase that targets Smad7 for ubiquitin degradation [36, 37]. It is reported that TGF-β1 induces Smurf2 expression via a Smad3-dependent mechanism [37]. Thus, the combination treatment with AA and NG produced a better inhibitory effect on Smad3 signaling via direct and indirect mechanisms.

We have previously shown that TGF-β/Smad3 mediates renal fibrosis by upregulating miR-21 but downregulating miR-29b in the UUO and diabetic nephropathy [38-41]. In the present study, we found that treatment with AA or NG also produced an additive effect on inhibition of miR-21 expression. However, treatment with AA and NG did not alter the expression of miR-29b *in vivo* and *in vitro*. Thus, inhibition of miR-21 expression may also account for a mechanism whereby AA, NG, and their combination treatment inhibited renal fibrosis.

The combination of AA and NG also allowed using lower dosages of AA and NG, which prevented the drug cytotoxicity while enhancing the therapeutic efficacy on renal fibrosis. Indeed, a single use of AA or NG induced an inhibition of Smad3 signaling in a dosage-dependent manner; however, it also caused the anti-proliferation effect and cytotoxicity when higher dosages were used. The combination of AA and NG allowed lowering the dosages of individual drugs to produce a safer but effective therapy for renal fibrosis without cytotoxicity. This is important since the cytotoxicity has been a major problem for the clinical application of the Traditional Chinese Medicine [42].

In summary, the present study demonstrates that AA functions as a Smad7 agonist and inhibits Smad3 signaling by inducing Smad7; whereas NG is a Smad3 inhibitor and blocks Smad3 signaling directly by inhibiting Smad3 phosphorylation and transcription. Thus, the combination of AA and NG produces an additive effect on inhibition of renal fibrosis by inhibiting Smad3 while upregulating Smad7 and may represent as a novel and effective therapy for chronic kidney disease including diabetic and hypertensive nephropathy.

## MATERIALS AND METHODS

### A mouse model of UUO and treatment

A mouse model of UUO was established in C57BL6 male mice (6 weeks of age, 20-22g) by ligating the left ureter as previously described [11]. Animals were randomly divided into 5 groups including Sham, UUO with vehicle control (DMSO) or treated with AA, NG, and the combination of AA plus NG. AA was a HLPC-purified product (95%) purchased from Guangxi Changzhou Natural Pharmaceutical Co. Ltd (Nanning, Guangxi, China). NG was obtained from Shanxi Huike Botanical Development Co. Ltd (Xian, Shanxi, China) with the HLPC-purity of 98%. Either AA or NG was dissolved in 1% DMSO and was administrated into mouse daily via intraperitoneal (i.p) injection. Briefly, after UUO surgery, groups of 8 mice were housed in the Animal Facility of Chinese University of Hong Kong (CUHK) with a 12/12-hour light/dark cycle and food and water available and received daily i.p. injections of control saline containing 1% DMSO, an optimal dose of AA at 5 mg/kg/boy weight (BW) and NG at 50 mg/kg/BW, or the combination of AA (5mg/kg/BW) plus NG (50 mg/kg/BW) for seven days. Kidney tissues were harvested and examined for renal fibrosis. The experimental procedures were approved by the Animal Ethics Committee of The CUHK.

### Determination of an optimal dose of AA, NG, and their combination ratio *in vitro* and *in vivo*

We first determined an optimal dosage of AA, NG, and their combination with the maximal inhibitory effect on TGF-β1/Smad signaling without cytotoxicity *in vitro* and *in vivo. In vitro*, a normal rat kidney tubular epithelial cell line (NRK52E) were cultured in Dulbecco's modified Eagle's medium (DMEM)-F12 medium supplemented with 5% fetal bovine serum (FBS). After being fasted, cells were incubated with AA (dissolved in 1% DMSO) at dosages of 0, 5, 10, 20, 30, 40, 50 μM and NG at dosages of 0, 25, 50, 100, 200, 400 μM for 24 hours. The supernatant from individual treatments was then collected for testing the cytotoxicity by the lactate dehydrogenase (LDH) releasing assay with a LDH release kit (BioVision Technologies, Exton, PA, USA) following the manufacturer's instructions. After determining safe dosages of AA and NG, a dose-dependent inhibitory effect of AA and NG on TGF-β/Smad signaling was performed in NRK52E cells with addition of a recombinant human TGF-β1 (2 ng/ml; R&D Systems, Minneapolis, MN, USA) for periods of 0, 30 min, 3h, 6h, and 24h following the 24-hour pre-incubation with the safe dosages of AA or NG. At least three-independent experiments were performed and 1% DMSO was used as control for all studies. Then, a safe and effective dose of AA and NG was selected for *in vitro* study.

Effective dosages of AA and NG for renal fibrosis were determined in groups of 4 mice with UUO by daily i.p injection of AA at dosages of 1.25, 2.5, 5.0, and 10 mg/kg body weight or NG at dosages of 12.5, 25, 50, and 100 mg/kg body weight for 7 days. A dose-dependent inhibitory effect of AA and NG on renal fibrosis was determined by western blot analysis of collagen I expression. After selecting an effective dosage for AA and NG, we performed a toxicity test *in vivo* in groups of 4 normal mice by a daily i.p. injection of DMSO, AA (5mg/kg BW), NG (50mg/kg BW), and their combination AA (5mg/kg/BW) plus NG (50 mg/kg/BW) for 7 days, respectively. Blood plasma was collected at day 7 after the treatment for the LDH releasing assay and 24-hour urinary samples were collected in the metabolism cages. Urinary albumin excretion (UAE) assay was performed by using competitive ELISA according to the manufacturer's instructions (Exocell, Philadelphia, PA, USA).

### Masson's trichrome staining and Immunohistochemistry

To evaluate the histological damage, collagen-like matrix deposition was stained with Masson's trichrome staining with the ‘Trichrome stain kit’ (Scy Tek Laboratoris, West Logan, UT) according to the manufacturer's instruction. Immunohistochemistry for detection of TGF-β/Smad signaling and renal fibrosis was performed in 4μm paraffin-embedded tissue sections of mouse kidney tissue using a microwave-based antigen retrieval technique [43]. Primary antibodies used in this study included rabbit polyclonal antibodies to TGF-β1, phosphorylated Smad2/3 (Santa Cruz Biotechnology, Santa Cruz, CA), collagen I (Southern Biotech, Birmingham, AL), α-SMA (Sigma, St. Louis, MO). The number of phospho-Smad2/3 was counted under high-power fields (×40) by means of a 0.0625-mm^2^ graticule fitted in the eyepiece of the microscope and expressed as cells per millimeters squared. The total collagen-like contents, expression of TGF-β1, and accumulation of collagen I and α-SMA on were measured in Masson's trichrome and immunostained sections by 10 random areas under high-power by using a quantitative image-analysis system (Image-Pro Plus 6.5, Media Cybernetics, Silver Spring, MD) and expressed as the percent positive area examined.

### RNA extraction and real-time PCR

Total RNA was isolated from kidney tissues and cultured cells using the RNeasy Isolation Kit (Qiagen, Valencia, CA) according to the manufacturer's instructions. Real-time PCR was performed using the Bio-Rad iQ SYBR Green supermix with Opticon2 (Bio-Rad, Hercules, CA) as previously described [16]. The primers used in the current study including mouse collagen I, α-SMA, TGF-β1, Smad7, Smurf2 and glyceraldehyde 3-phosphate dehydrogenase (GAPDH) were described previously [35, 44]. In addition, expression of miR-21 and miR-29b expression in cultured TECs and in the UUO kidney was measured by real-time PCR with primers as previously described [38, 39]. The ratio for the mRNA of interest was normalized to GAPDH and expressed as the mean ± standard errors of the mean (SEM).

### Western blot analysis

Proteins from kidney tissues and cultured cells were extracted with RIPA lysis buffer, and western blot analysis was performed as described previously [10]. After blocking the nonspecific binding with 5% BSA (1h, room temperature), membranes were then incubated with the primary antibody against phospho-Smad3 (Cell Signaling Technology), collagen I (Southern Biotech), Smad7, Smurf2, total Smad3 (Santa Cruz Biotechnology), α-SMA, glyceraldehyde 3-phosphate dehydrogenase (Chemicon, Temecula, CA) overnight at 4°C, followed by the IRDye 800-conjugated secondary antibody (Rockland immunochemicals, Gilbertsville, PA). Signals were captured using the LiCor/Odyssey infrared image system (LI-COR Biosciences, Lincoln, NE) and the intensity of each band was quantified and analyzed by using the Image J software (NIH, Bethesda, MD, USA).

### Urinary albumin excretion

To determine the potential toxicity to the kidney, 24-hour urinary samples from groups of 4 normal mice were collected in the metabolic cages after treatment with AA, NG, and their combination for 7 days. Urinary albumin excretion was measured by competitive ELISA according to the manufacturer's instructions (Exocell, PA). Urinary creatinine was measured by an enzymatic kit (Stanbio Labs, TX). Urinary albumin excretion was expressed as total urinary albumin/creatinine ratio.

### Statistical analyses

Data are expressed as the mean ± SEM and analyzed using one-way analysis of variance (ANOVA), followed by Tukey post-hoc tests using GraphPad Prism 5 (GraphPad Software, San Diego, CA).
